# Great blue-shift of luminescence of ZnO nanoparticle array constructed from ZnO quantum dots

**DOI:** 10.1186/1556-276X-6-338

**Published:** 2011-04-14

**Authors:** Nengwen Wang, Yuhua Yang, Guowei Yang

**Affiliations:** 1 State Key Laboratory of Optoelectronic Materials and Technologies, Institute of Optoelectronic and Functional Composite Materials, Nanotechnology Research Center, School of Physics & Engineering, Sun Yat-sen University, Guangzhou 510275, Guangdong, P. R. China

## Abstract

ZnO nanoparticle array has been fabricated on the Si substrate by a simple thermal chemical vapor transport and condensation without any metal catalysts. This ZnO nanoparticles array is constructed from ZnO quantum dots (QDs), and half-embedded in the amorphous silicon oxide layer on the surface of the Si substrate. The cathodoluminescence measurements showed that there is a pronounced blue-shift of luminescence comparable to those of the bulk counterpart, which is suggested to originate from ZnO QDs with small size where the quantum confinement effect can work well. The fabrication mechanism of the ZnO nanoparticle array constructed from ZnO QDs was proposed, in which the immiscible-like interaction between ZnO nuclei and Si surface play a key role in the ZnO QDs cluster formation. These investigations showed the fabricated nanostructure has potential applications in ultraviolet emitters.

## Introduction

Recently, ZnO has attracted very great attention because of its particular properties in broad fields. For example, it has a large direct band gap of 3.37 eV and exciton-binding energy of 60 meV, while the Bohr radius of exciton is as small as approx. 2.34 nm. Thus, ZnO is a promising candidate for the high efficient ultraviolet (UV) laser device [1-3]. Interestingly, when the size of ZnO nanoparticles is smaller than the Bohr radius (i.e., ZnO quantum dots, QDS), the quantum confinement has a notable influence on the band gap and further causes a series of novel characteristics such as the blue-shift of luminescence [4-7]. Therefore, there have been a variety of techniques to fabricate ZnO QDs [6-10]. Usually, the size of ZnO QDs is slightly larger than or just comparable with the exciton Bohr radius [8-13]. However, few research reports have been involved in the ZnO QDs, showing that their dimension is rigorously smaller than the Bohr radius [4-13].

In this study, we have fabricated the unique ZnO nanoparticle arrays that are constructed from ZnO QDs blocks on silicon substrates using a simple thermal chemical vapor transport and condensation without any metal catalysts. Importantly, we measure a great blue-shift of luminescence in the cathodoluminescence (CL) spectrum of the fabricated nanostructure, which implies that this ZnO QDs structure would be applicable to optoelectronic and spintronic applications.

## Experimental

The ZnO nanoparticle array is fabricated by a simple thermal vapor transport method, and the detailed experimental process has been reported in our previous study [13]. Simply, Si wafers serving as substrates are loaded downstream in a quartz tube. Zinc oxide powders and graphite powders are mixed and heated to 1050°C under the argon gas flow at the rate of 50 sccm with a pressure of 9.0 × 10^4 ^Pa. Half-an-hour later, the source powders and the substrate are all taken out from the furnace and allowed to cool down to room temperature naturally. Field emission scanning electron microscopy (FESEM), X-ray diffraction (XRD), and transmission electron microscopy (TEM) coupled with electron-energy loss spectroscopy (EELS) are employed to characterize the morphologies and structures of the prepared samples. The CL measurement is carried out at room temperature using a Gatan Mono-CL system coupled to FESEM with the accelerating voltage of 10 kV.

## Results and discussion

**Figure 1:**
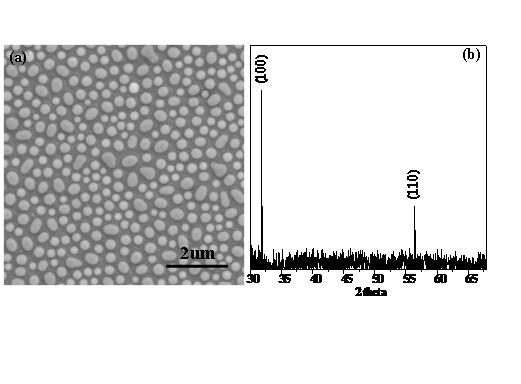
**The feature and structure of the prepared sample**. SEM image (a) and corresponding XRD pattern (b) of the fabricated ZnO nanoparticle array.

The fabricated nanoparticle array is shown in Figure 1. Clearly, these nanoparticles are relatively uniform in size and array, isolated, and elliptical. They are half-embedded in the surface of the Si substrate. The corresponding XRD pattern (Figure 1b) can be indexed to be the wurtzite ZnO structure with (100) and (110) peaks. Therefore, these results show that the prepared nanoparticles are ZnO. Note that we can control the size of the fabricated nanoparticles by the growth conditions such as the growth time.

**Figure 2:**
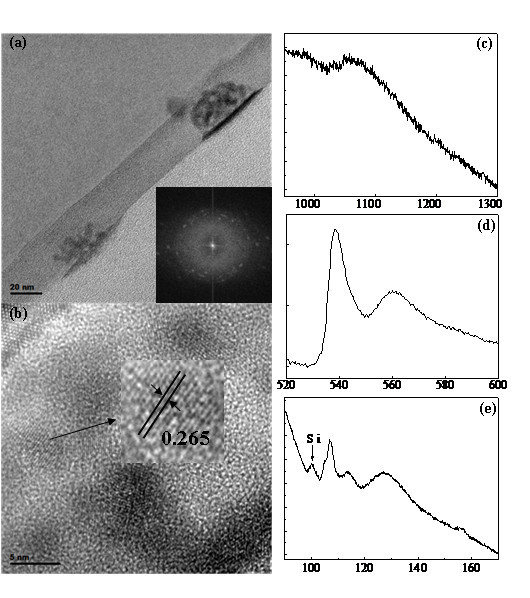
**The TEM and EELS analysis of the structure details of the sample**. TEM image with the inserted FFT pattern of the sample in a large area (a), HRTEM image with a highlighted ZnO nanoparticle and the corresponding interplanar spacing (b), EELS of the Zn-L edge (c), O-K edge (d), and Si-L edge (e).

In order to verify the detailed structure of the fabricated nanoparticle array, we prepare the cross-sectional sample for TEM characterization, and the results are shown in Figure 2. In the low magnification of TEM image in Figure 2a, the thickness of the layer is uniform of approx. 25 nm, while two high contrast particles are implanted in the layer. The sizes of the two particles are, respectively, 50 and 57 nm at the interface. The FFT pattern (the inset of Figure 2a) of one particle indicates that it is polycrystalline. The HRTEM image in Figure 2b is taken from the upper ZnO nanoparticle in Figure 2a. Clearly, we can see that several small crystalline particles gather together and form one nanoparticle. The average size of these small ZnO particles is 5.5 nm, which are the so-called ZnO QDs [4-13]. One ZnO QD has been emphasized and marked with the interplanar spacing of 0.265 nm in the inset of Figure 2b, which is corresponding to the plane (002) of the wurtzite ZnO. Actually, all the interplanar spacings of QDs in Figure 2b and other HRTEM data can be assigned to the spacings of the wurtzite ZnO structure. In addition, we can easily observe that these ZnO QDs are embedded in the amorphous silicon oxide layer on the surface of the Si substrate. Therefore, these results show that the fabricated ZnO nanoparticle array is constructed from ZnO QDs.

The EELS spectra of the Zn-L, O-K, and Si-L edges on the particle zone of the sample exhibited in Figure 2c,d,e show that the ZnO nanoparticles contain Zn, O, and Si elements. The sets of Zn-L edge with the peak centered at 1050 eV and the O-K edge with the feature peak at 538 eV demonstrate that the nanoparticles are zinc oxide, in accordance with reports and the analytic results shown above, while the spectra shift due to the native defects, such as Zn and O vacancies on the surface of ZnO QDs [14-17]. As we see the Si-L edge in Figure 2e, the distinct features are at 100, 107, 114, 127, and 157 eV, respectively. This Si-L edge is very similar with the spectrum of silicon monoxide that is overlapped by spectra of elemental silicon and of SiO_2 _whose onsets of the L_2,3_-edge are approx. 100 and 107 eV, respectively [18-23]. Thus, these results reveal that the ZnO QDs disperse in the silicon monoxide.

**Figure 3:**
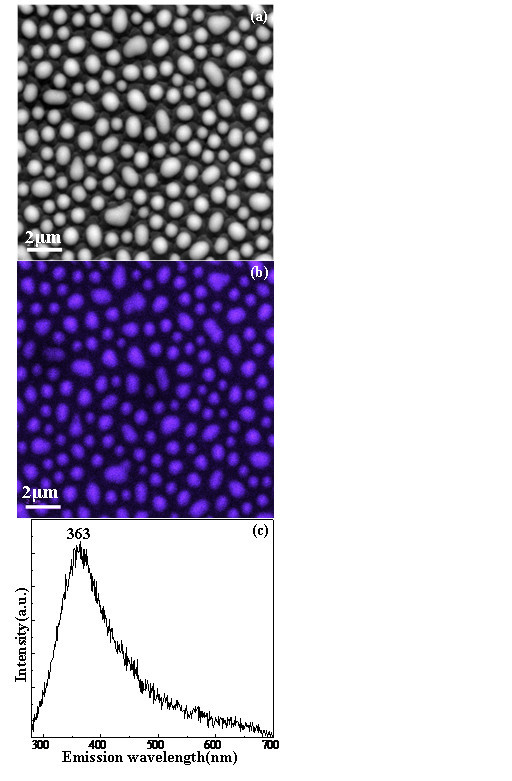
**The CL measurements of the ZnO nanoparticle array**. SEM image (a), the panchromatic CL image (b), and the corresponding CL spectrum (c).

**Figure 4:**
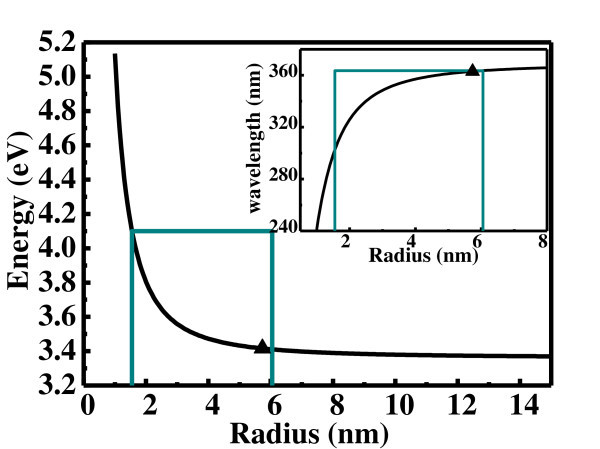
**The dimension of QDs dependence of band gap according to formula (1) and the inset of the relevant emission wavelength dependent on the dimension of QDs**. The triangle spot signifies the energy and wavelength which are related to the experimental peak of 363 nm.

In order to explore this fabricated nanostructure's potential applications, we measure the optical properties as shown in Figure 3. Figure 3 shows the CL measurement of the sample. The panchromatic CL image in Figure 3b exhibits that the intense luminescence is mainly from the ZnO nanoparticles. Interestingly, we can observe that the luminescence peak is centered at 363 nm as shown in Figure 3c, which is known as the near-band edge emission of ZnO. However, there is a great blue-shift compared to bulk ZnO in the CL spectrum. Based on previous reports [1,10,24,25], the blue-shift of the CL spectrum of ZnO QDs in our studies is attributed to the quantum-size confinement effect as follows [5,26](1)

where *ћ *is the Planck's constant, *R *is the radius of ZnO QDs,  and  are, respectively, the effective masses of electron and hole (taking  and [27]), *E*_(gap, bulk) _is the bulk ZnO band gap (3.377 eV), and  is the exciton-binding energy (60 meV [2]). Based on Equation 1, we can obtain the relationship between the size and band gap of ZnO QDs as shown in Figure 4. In our case, the radius of ZnO QDs is in the range of 1.6-6.1 nm are also shown in Figure 4. The corresponding band gap and emission wavelength ranges of the prepared ZnO QDs with the radius of 1.6-6.5 nm are also shown in Figure 4. Meanwhile, the peak of 363 nm in the CL spectrum in Figure 3c is corresponding to the size of 5.7 nm for ZnO QDs according to Equation 1. Therefore, the experimental observations are consistent with the theoretical values in our studies. These results thus show that the great blue-shift compared to bulk ZnO is attributed to the quantum size confinement. However, the theoretical emission peak of ZnO QDs with the radius in 1.6-6.1 nm seems about 340 nm that is corresponding to the average radius of the fabricated ZnO QDs in our case based on Equation 1. In fact, the experimental peak actually shifts to the low energy or high wavelength in Figure 4. As we know, the intensity of emission of big QDs is stronger than that of small QDs. Therefore, the emission from big QDs is easily measured in experiment, which cases the measured emission peak shifting to the low energy or the high wavelength as shown in Figure 4.

**Figure 5:**
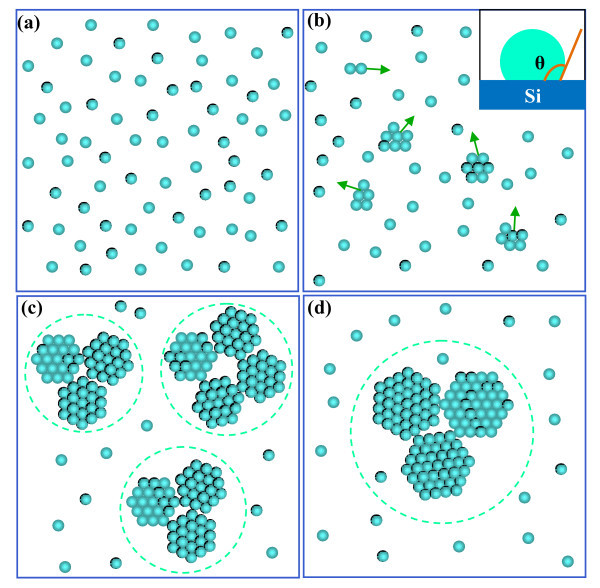
**Schematic illustration of the fabrication mechanism of the ZnO nanoparticle array constructed from ZnO QDs**. ZnO molecules randomly diffusing on surface (a), ZnO clusters thermally diffusing on surface and the inset showing the contact angle θ (b), large clusters formation by small clusters continuously diffusing and colliding (c), and big particles formation (d).

According to our previous study [28,29], the fabrication mechanism of the nanoparticle array is suggested a vapor-solid process. First, ZnO molecules form the thermal chemical vapor transport of source deposit on the substrate and then thermally diffuse on surface. Second, many small ZnO clusters would form by ZnO molecules by ZnO molecules continuously diffusing and colliding as shown in Figure 5b. Then, these small ZnO clusters still thermally diffuse on the surface, because there is an immiscible-like interaction between ZnO cluster and Si surface. In the inset in Figure 5b, we can see that the contact angle between ZnO cluster and Si surface is about 110° [28-33]. Thus, this contact angle is so large that ZnO clusters could easily thermal diffuse on Si surface, which seems a driving force to push ZnO cluster moving on surface. Third, large ZnO clusters would form by small clusters continuously diffusing and colliding as shown in Figure 5c. Actually, the nucleation of ZnO could take place when the size of clusters reaches to that of the critical nucleus in this stage. Then, these small ZnO nuclei still thermally move on surface because of the immiscible-like interaction between ZnO cluster and Si surface. Finally, these particle constructed from small nuclei would stop moving on surface and grow up step by step when their size is sufficiently large as shown in Figure 5d. In other words, the large cluster will stand on surface when the immiscible-like interaction cannot provide sufficiently large driving force to push those big particles. In addition, Si surrounding ZnO QDs would be oxidized to form silicon oxides. Thus, we can see that the ZnO nanoparticles are half-embedded in the amorphous silicon monoxide.

## Conclusion

In summary, we have fabricated the ZnO nanoparticle array which is constructed from ZnO QDs on the Si substrate by the thermal chemical vapor transport and condensation without any metal catalysts. This fabricated ZnO nanostructure exhibited a great blue-shift of luminescence in the CL spectrum. These novel properties show that the ZnO nanoparticle array has potential applications in UV emitters.

## Abbreviations

CL: cathodoluminescence; EELS: electron-energy loss spectroscopy; FESEM: field emission scanning electron microscopy; QDs: quantum dots; TEM: transmission electron microscopy; UV: ultraviolet; XRD: X-ray diffraction.

## Competing interests

The authors declare that they have no competing interests.

## Authors' contributions

N. W. Wang carried out the analysis and study of ZnO nanoparticle arrays, participated in the sequence alignment and drafted the manuscript. Y. H. Yang participated the luminescence analysis. G. W. Yang conceived of the study and participated in its design, coordination and the sequence alignment. All authors read and approved the final manuscript.
